# The Dampening Effects of Perceived Teacher Enthusiasm on Class-Related Boredom: The Mediating Role of Perceived Autonomy Support and Task Value

**DOI:** 10.3389/fpsyg.2017.00400

**Published:** 2017-03-17

**Authors:** Guanyu Cui, Meilin Yao, Xia Zhang

**Affiliations:** ^1^Institute of Developmental Psychology, School of Psychology, Beijing Normal UniversityBeijing, China; ^2^Department of Psychology, Henan Medical UniversityZhengzhou, China; ^3^Department of Nursing, Henan Medical UniversityZhengzhou, China

**Keywords:** class-related boredom, teacher enthusiasm, task value, autonomy support, mediating role

## Abstract

Class-related boredom is commonly experienced by students and it has an impact on their learning engagement and achievements. Previous research has found that perceived teacher enthusiasm might contribute to reducing students’ class-related boredom. However, the mechanism through which perceived teacher enthusiasm affects class-related boredom remains unexplored. The purpose of the present study was to investigate the mediating role of perceived autonomy support and task value in the relationship between teacher enthusiasm and class-related boredom. College students (*N* = 734) completed questionnaires on perceived teacher enthusiasm, boredom proneness, perceived task difficulty, perceived autonomy support, perceived task value, and class-related boredom. Results showed that after controlling for the effects of demographic variables, boredom proneness, and perceived task difficulty, both perceived autonomy support and task value fully mediated the relationship between perceived teacher enthusiasm and class-related boredom. These findings suggest that students who perceive more teacher enthusiasm might perceive more autonomy support and task value, which in turn reduce the students’ class-related boredom. Limitations in the present study have also been discussed.

## Introduction

As a type of “user experience,” class-related boredom is a common emotion experienced by students in various school settings. It was found that low perceived value of class-related tasks might be a major cause of students’ boredom (Pekrun, 2006; Pekrun et al., 2007, 2010). Therefore, strategies to increase task value and reduce the level of boredom in classroom settings have attracted the attention of researchers and educators (Perkins and Hill, 1985; Larson and Richards, 1991; Pekrun et al., 2010). Enthusiastic teaching behavior was conceived as an important environmental factor for improving the task value perceived by students (Hatfield et al., 1994; Pekrun, 2006; Pekrun et al., 2007; Frenzel et al., 2009). Previous studies primarily focused on the behavioral aspects of teacher enthusiasm and their effects on students’ learning motivation, achievement, and positive emotions (Keller et al., 2015). However, few studies have explored the mechanism through which teacher enthusiasm affects students’ class-related emotions, especially regarding negative emotions such as class-related boredom (Goetz et al., 2013; Keller et al., 2014). Furthermore, the level of teacher enthusiasm perceived by students may play a more important role in their learning outcomes as compared to that reported by the teachers themselves (Keller et al., 2014, 2015). However, the question of whether and how perceived teacher enthusiasm can significantly predict class-related boredom remains unclear.

### Theories about the Relationship between Teacher and Student Emotions

Previous research has used the emotion contagion theory (Barsade, 2002; Keller et al., 2014) and emotional crossover theory (Becker et al., 2014, 2015) to explain the direct effects of teachers’ emotions on students’ emotions in the classroom. However, researchers have found that the paths were more complex than a simple direct association. Over the past decade, Pekrun’s control-value theory of achievement emotions has become one of the most well-known theories in the domain of achievement emotions (Pekrun, 2006; Pekrun et al., 2007). Pekrun’s theory provided a background for exploring the mechanism through which teacher emotions affect students’ learning outcomes.

According to the theoretical framework of the control-value theory of achievement emotions, teacher enthusiasm is a component of value induction and can affect students’ achievement emotions through the mediation of the control and values perceived by students (Pekrun, 2006; Pekrun et al., 2007). Regarding boredom, according to Pekrun’s integral theoretical framework, high/low control may cause boredom, while appropriate control may not do so. Additionally, perceived valuelessness was one of the most important antecedents of boredom (Pekrun, 2006; Pekrun et al., 2007). Teacher enthusiasm (corresponding to value induction in the control-value theory of achievement emotions) may have positive effects on students’ perceived task value which in turn may have major effects on reducing student boredom. Based on the control-value theory of achievement emotions, the present study focused on the mediating role of task value between teacher enthusiasm and students’ class-related boredom. Given that the study by Kunter et al. (2008) found that teachers’ enthusiasm for teaching predicted students’ perceived social support, we aimed to investigate the mediating roles of perceived autonomy support and task value in the relationship between students’ perceived teacher enthusiasm and class-related boredom. Considering the possible influences of boredom proneness and task difficulty (corresponding to control in the control-value theory of achievement emotions) on class-related boredom (Mann and Robinson, 2009), we aimed to control for these two variables (i.e., boredom proneness and task difficulty) and focused mainly on the mechanism through which students’ perceived teacher enthusiasm affects their class-related boredom.

There may be differences between students’ perceived teacher enthusiasm and teachers’ self-reported enthusiasm. In recent years, researchers have begun to pay more attentions to students’ perceptions of the classroom environment and its effect on the learning process (Marsh, 2007; Marsh et al., 2009, 2012; Fauth et al., 2014). Individual students’ perceptions of their classroom environment were related to their learning outcomes, and therefore, could be used as reliable indicators at the individual level (Lüdtke et al., 2009; Keller et al., 2014, 2015). Thus, in the current study, we focused on perceived classroom environment and learning process at the individual level.

### Class-Related Boredom

Academic boredom is one of the most widespread emotions experienced by students in the framework of academic emotions, and it can be classified into class-related boredom and learning-related boredom (Pekrun et al., 2002, 2010). Class-related boredom is a type of state boredom experienced by students in the course of class activities (Pekrun et al., 2010). Class-related boredom functions at a higher level than learning-related boredom does, as experienced by students (Tze et al., 2015). The boredom experienced by students can provide important information, such as that regarding the working or learning environment, or that when seeking to prevent excessive involvement in uninteresting tasks or the generation of severe psychological problems (Elpidorou, 2014). Furthermore, many studies found that class-related boredom had several negative effects on academic performance and health. For example, class-related boredom experienced frequently or for a long time may result in a relatively stable bored belief or trait boredom, which may affect school learning, career choices (Watt and Vodanovich, 1999; Wigfield et al., 2002), and lifelong learning (Goetz et al., 2003) in relevant domains. A recent meta-analysis by Tze et al. (2015) investigated the relationship between boredom and academic outcomes. Their results showed that boredom has negative effects on learning motivation, the use of learning strategies, and achievement.

### Perceived Teacher Enthusiasm

Teacher enthusiasm has been regarded as one of the most important teaching qualities and class-related environmental factors (Locke and Woods, 1982; Brophy and Good, 1986; Patrick et al., 2000; Long and Hoy, 2006; Kunter et al., 2008, 2011, 2013; Keller et al., 2013, 2014, 2015). Despite the long history of research on teacher enthusiasm in educational psychology, earlier studies focused mainly on teachers’ external behaviors in the course of teaching, such as voice, tone, facial expression, and body posture (i.e., gestures) (Brophy and Good, 1986). Later, Kunter et al. (2008) asserted that teacher enthusiasm expressed by external behaviors may not be consistent with teachers’ own internal and experienced affect, and further research should be conducted to reveal stable and authentic teacher enthusiasm. Kunter et al. (2008, 2011) classified teacher enthusiasm into two categories: enthusiasm for the subject and enthusiasm for teaching. More recently, Keller et al. (2014, 2015) proposed an integrated teacher enthusiasm construct. Keller et al. (2014) found that the new construct of integral teacher enthusiasm affected students’ interest in learning through the full mediation of perceived teacher enthusiasm. Thus, students’ perceived teacher enthusiasm may provide more direct and rich information about the relationship between teacher enthusiasm and students’ outcomes.

### Dampening Effects of Perceived Teacher Enthusiasm on Class-Related Boredom

Abundant empirical studies have found that teacher enthusiasm positively affected students’ learning outcomes (Patrick et al., 2000; Long and Hoy, 2006; Kunter et al., 2008, 2011, 2013; Keller et al., 2013, 2014, 2015). Studies by Kunter et al. (2008, 2011, 2013) and Keller et al. (2013, 2014, 2015) found that teacher enthusiasm correlated with the high quality of teaching and students’ positive learning outcomes (e.g., enjoyment in learning). Frenzel et al. (2009), Kim and Schallert (2014) found that perceived teacher enthusiasm could predict students’ learning enjoyment and interest. Additionally, studies revealed that students’ interest in learning was a negative predictor of boredom (Daschmann et al., 2014), and interest in specific tasks was negatively correlated with boredom (Tanaka and Murayama, 2014). Based on the separated and reciprocally related, yet not mutually exclusive, relationships between positive and negative emotions (Cacioppo and Berntson, 1994; Schimmack, 2001; Smith et al., 2006; Schimmack and Colcombe, 2007), perceived teacher enthusiasm might induce situational interest (Kim and Schallert, 2014) and reduce class-related boredom (Goetz et al., 2006). Accordingly, we could infer that perceived teacher enthusiasm may negatively predict class-related boredom.

### Mediating Model

The control-value theory of achievement emotions proposed an integral framework of achievement emotions with their antecedents and outcomes (Pekrun, 2006; Pekrun et al., 2007). In this theoretical framework, instruction, value induction, and autonomy support were identified as important environmental variables that may affect various achievement emotions (both activity emotions and outcome emotions) through perceived control and value as mediators. One of the components of value induction, i.e., teacher enthusiasm, was conceived to be a facilitator of task value. Therefore, teacher enthusiasm may affect emotions through the mediation of perceived task value (Pekrun, 2006; Pekrun et al., 2007). Autonomy support was an important environmental variable in the theoretical framework; however, the relationships between the specific components of value induction, perceived task value, discrete emotions, and autonomy support have not been addressed. For example, the relationships among teacher enthusiasm, autonomy support, perceived task value, and class-related boredom are yet to be explored in depth. Additionally, the theoretical framework did not indicate whether various environmental variables could predict discrete emotions through different mediating paths (Pekrun, 2006; Pekrun et al., 2007). Recent quantitative results did not support particular hypotheses in the theoretical framework, for example, achievement goals did not affect academic achievement through the mediation of achievement emotions (Lüftenegger et al., 2016). Therefore, further studies need to be conducted to examine the theoretical framework.

### Perceived Autonomy Support as a Mediator

The teachers’ autonomy support that is perceived by students is an important perceived environmental variable in the classroom. Teachers’ autonomy support was derived from the self-determination theory (SDT), and it refers to teachers’ behaviors such as providing choices, encouraging autonomy, listening to students, and understanding the feelings of students (Deci and Ryan, 1987; Deci et al., 1991).

Enthusiastic teachers may provide students with more autonomy support, which in turn may be perceived by students. Based on the SDT (Deci and Ryan, 1987; Deci et al., 1991), we hypothesized that enthusiastic teachers may fulfill their psychological needs in the course of teaching, learning them to experience more positive emotions and feelings. Furthermore, as an internal incentive, these positive emotions and feelings may facilitate teachers to provide more autonomy support for their students during teaching. This hypothesis was supported by empirical studies. Earlier research showed that enthusiastic teachers might provide more autonomy support for students or they may exert less personal control over them (Rosenshine, 1970). Research by Kunter et al. (2008) showed that teachers with more enthusiasm for mathematics could provide more cognitive autonomy support for students. At the same time, teachers with more enthusiasm for teaching could provide more social support for their students. Studies on intrinsic motivation showed that teachers who experienced pleasure and internal incentives provided more support for their students, ultimately facilitating students’ learning motivation (Roth et al., 2007; Klusmann et al., 2008; Kunter et al., 2008; Frenzel et al., 2009). Generally, teacher enthusiasm and autonomy support are core components of the classroom learning environment, which may have potential influences on students. Therefore, we hypothesized that students who perceived more teacher enthusiasm may also perceive more autonomy support from their teachers.

Students’ perceived autonomy support may reduce their class-related boredom. Previous research showed that perceived autonomy support positively affected students’ learning outcomes (e.g., Reeve et al., 2004; Tsai et al., 2008; Sierens et al., 2009; Jang et al., 2010; Sakiz, 2012). For example, Tsai et al. (2008) found that perceived autonomy support among Grade -7 students positively predicted their interest in mathematics. Sierens et al. (2009) found that if teachers did not provide solid autonomy support, undergraduate students were less likely to achieve high levels of self-regulation, in spite of structured instruction and clear expectations. Additionally, some study results showed that autonomy support was negatively and significantly correlated with negative emotions (Daschmann et al., 2011; Kaplan and Assor, 2012). Especially, Tze et al. (2014) found that perceived autonomy support was negatively associated with class-related boredom. Therefore, we hypothesized that students’ perceived autonomy support could negatively predict class-related boredom.

In summary, based on the results of prior studies, we hypothesized that perceived autonomy support might mediate the relationship between perceived teacher enthusiasm and class-related boredom.

### Perceived Task Value as a Mediator

On the one hand, teacher enthusiasm may facilitate task value. According to the emotional contagion theory (Barsade, 2002), teacher enthusiasm leads to increased positive emotions and perceived task value by students. Based on the social cognitive theory of learning (Bandura, 1977; Pekrun, 2000) and the theory of social constructivism (Wild et al., 1992, 1997), students’ perceived teacher enthusiasm toward a subject and teaching is considered to affect their perception and evaluation of task value. New empirical research conducted by Keller et al. (2014) found that perceived teacher enthusiasm significantly predicted students’ learning value (included in the construct of individual interest). On the other hand, according to the control-value theory of achievement emotions, students’ perceived valuelessness is an important contributor to boredom (Pekrun, 2006; Pekrun et al., 2007). Therefore, we hypothesized that perceived task value might play a mediating role between perceived teacher enthusiasm and class-related boredom.

### Perceived Autonomy Support and Perceived Task Value as Mediators

Students’ perceived autonomy support may facilitate their perceived task value and may reduce their class-related boredom. Based on the SDT, the autonomy support perceived by students may fulfill their psychological needs, which may facilitate their perceived task value, interest, learning motivation, and achievement (Deci et al., 1991). According to the control-value theory of achievement emotions, autonomy support affects emotions and learning outcomes through the mediation of perceived control and value (Pekrun, 2006). Patall et al. (2013) found that provision of choice (a core component of autonomy support) was related to greater course value perceived by students. Therefore, we hypothesized that perceived task value might mediate the relationship between perceived autonomy support and class-related boredom.

Perceived choice, a core component of perceived autonomy support, was confirmed to lead to individual perceptions of autonomy and sense of competence, and in turn, it was considered to affect individuals’ motivation and performance outcomes (Patall et al., 2008, 2010, 2014; Patall, 2012, 2013). Furthermore, Ruzek et al. (2016) found that perceived autonomy mediated the relationship between teacher emotional support, and students’ engagement and motivation (Ruzek et al., 2016). According to the integral framework of the control-value theory of achievement emotions, teacher enthusiasm is one of the components of value induction (Pekrun, 2006; Pekrun et al., 2007). Therefore, we hypothesized that perceived autonomy support may mediate the relationship between teacher enthusiasm and students’ perceived task value. Combining the above two hypotheses, we concluded that both perceived autonomy support and perceived task value might mediate the relationship between teacher enthusiasm and class-related boredom in serial paths. That is, students may perceive more autonomy support and task value from their enthusiastic teachers, and they may experience lower class-related boredom.

In summary, it is necessary to explore the dampening effects of perceived teacher enthusiasm on class-related boredom and to reveal the mechanism behind these effects. Few studies on teaching and learning have addressed whether perceived teacher enthusiasm can reduce the level of negative emotions (e.g., class-related boredom). The present study aimed to explore the positive effects of teacher enthusiasm on a wider range of class-related emotions as well as on learning outcomes, and to reveal the mediating role of perceived autonomy support and task value.

### Overview of the Current Study

Based on the above literature review, we proposed the following four hypotheses. Hypothesis 1: Perceived teacher enthusiasm has a negative effect on class-related boredom; Hypothesis 2: Perceived autonomy support plays a mediating role in the relationship between perceived teacher enthusiasm and class-related boredom; Hypothesis 3: Perceived task value plays a mediating role in the relationship between perceived teacher enthusiasm and class-related boredom; and Hypothesis 4: Perceived teacher enthusiasm can significantly predict class-related boredom through the serial mediating role of perceived autonomy support and perceived task value.

**FIGURE 1:**
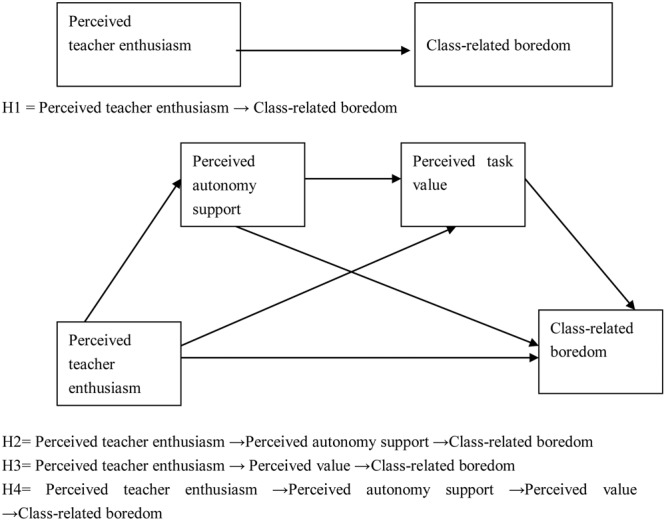
**Mediation model**.

To test these hypotheses, we constructed a hypothetical multiple mediation model to investigate whether perceived teacher enthusiasm could dampen college students’ class-related boredom via students’ perceived autonomy support and task value (see **Figure 1**).

## Materials and Methods

### Participants and Procedures

The Research Ethics Committee of School of Psychology, Beijing Normal University. The survey was conducted with 734 (91.6% female) college students majoring in clinical medicine, nursing, pharmacy, or medical technology, with a mean age of 19 years (*SD* = 1.09 years). Prior to participation, students were informed about the goals of the study, duration, procedure, and confidentiality of their data. Participation in the study was voluntary, informed consent was assured, and students did not receive compensation for their participation. Participants were assured that all of their responses would remain confidential and would not influence their course grade. All students were asked to evaluate their class-related feelings toward courses including “basic nursing science,” “normal human tissue and anatomy,” “medical nursing,” “diagnostics,” “cosmetic technique,” “biochemistry,” “organic chemistry,” and others.

### Measures

#### Boredom Proneness Scale-Short form (BPS–SF)

Boredom proneness was measured using the BPS-SF, a 12-items self-report instrument (Vodanovich et al., 2005). According to Huang et al. (2010) and the current study, two items were deleted (i.e., “I find it easy to entertain myself” and “It seems that the same old things are on television or the movies all the time; it’s getting old”), as they did not fit a Chinese college student model according to a confirmatory factor analysis; thus, 10-items were ultimately used. Two example questions are, “It is easy for me to concentrate on my activities” and “Many things I have to do are repetitive and monotonous.” Responses are indicated on a seven-point Likert scale ranging from 1 (strongly disagree) to 7 (strongly agree). A higher aggregate score indicates a higher level of boredom proneness. The Cronbach’s alpha was 0.65 for this scale in the present study.

#### Perceived Task Difficulty

Two items (i.e., “Today’s class was hard for me” and “Compared to other courses, today’s class was hard for me”) from the studies by Eccles and Wigfield (1995), Wigfield and Eccles (2000), and Tanaka and Murayama (2014) were used to assess perceived task difficulty. Responses to both items are indicated on a five-point Likert scale ranging from 1 (not at all true of me) to 5 (very true of me). A higher aggregate score indicates a higher level of perceived task difficulty. The Cronbach’s alpha was 0.77 for this measure in the present study.

#### Perceived Teacher Enthusiasm

Three items (i.e., “Our teacher in this class teaches with enthusiasm,” “Our teacher in this subject enjoys teaching compared to other courses,” and “Our teacher in this class tries to inspire students about the subject”) from the study by Keller et al. (2014) was used to assess perceived teacher enthusiasm. Responses are indicated on a five-point Likert scale ranging from 1 (not at all true of me) to 5 (very true of me). A higher aggregate score indicates higher levels of perceived teacher enthusiasm. The Cronbach’s alpha was 0.85 for this measure in the present study.

#### Perceived Autonomy Support

The Learning Climate Questionnaire (LCQ) was used to assess perceived autonomy support (Williams and Deci, 1996). This questionnaire has been widely used to assess perceived autonomy support in classroom investigations (Filak and Sheldon, 2008; Zhou et al., 2009; Reeve, 2013; Tze et al., 2014). According to Chen and Guo (2014) and the current study, two items were deleted (i.e., “I am able to be open with my instructor during class” and “I don’t feel very good about the way my instructor talks to me”), as they did not fit a Chinese college student model as per the confirmatory factor analysis; therefore, 13-items were used. Two example questions are, “I feel that my instructor accepts me” and “My instructor answers my questions fully and carefully.” Responses are indicated on a five-point Likert scale ranging from 1 (not at all true of me) to 5 (very true of me). A higher aggregate score indicates a higher level of perceived autonomy support. The Cronbach’s alpha for the 13-items scale was 0.94 in the present study.

#### Perceived Task Value

Two items (i.e., “What I learned in today’s class was useful” and “Compared to what I studied in other courses, what I studied in today’s class was useful”) from the studies by Eccles and Wigfield (1995), Wigfield and Eccles (2000), and Tanaka and Murayama (2014) were used to assess perceived task value. Responses to both items are indicated on a five-point Likert scale ranging from 1 (not at all true of me) to 5 (very true of me). A higher aggregate score indicates a higher level of perceived task value. The Cronbach’s alpha of this scale was 0.76 in the present study.

#### Class-Related Boredom

Eleven items from the class-related boredom scale included in the Achievement Emotions Questionnaire (AEQ) were used to assess college students’ class-related boredom in this study (Pekrun et al., 2005, unpublished). Two example questions are “The lecture bores me” and “I think about what else I might be doing rather than sitting in this boring class.” Responses are indicated on a five-point Likert scale ranging from 1 (strongly disagree) to 5 (strongly agree). A higher aggregated score indicates a higher level of class-related boredom. The Cronbach’s alpha for the 11-item tool was 0.94 in the current study.

### Statistical Analyses

Firstly, we examined the descriptive statistics and intercorrelations of the study variables using PASW statistics for Windows (Version 18, IBM, Corp., Armonk, NY, USA), and the mean, standard deviation, and intercorrelations of the sample and related variables were obtained. Subsequently, we examined the pattern of relationships in our theoretical model through a path analysis using Mplus 7 (Muthén and Muthén, 1998–2013). The path analysis was used to test the direct and indirect relationships among variables, which can provide estimates of the magnitude and significance of the causal connections hypothesized between variables. The BC bootstrap method with 1000 bootstrap samples was selected to confirm the significance of the mediating effects of perceived autonomy support and task value on the link between perceived teacher enthusiasm and class-related boredom experienced by students. This method is included as an option in Mplus and it produces the most accurate confidence limits with the largest power for detecting mediation effects (Cheung and Lau, 2008).

## Results

### Descriptive Analyses

**Table 1** shows the means, standard deviations, and intercorrelations of the dependent variables in this study: boredom proneness, perceived task difficulty, perceived teacher enthusiasm, perceived autonomy support, and perceived task value (*n* = 734). To be more specific, class-related boredom was positively related to boredom proneness (*r* = 0.37, *p* < 0.001) and perceived task difficulty (*r* = 0.28, *p* < 0.001) but negatively related to perceived teacher enthusiasm (*r* = -0.26, *p* < 0.001), perceived autonomy support (*r* = -0.40, *p* < 0.001), and perceived task value (*r* = -0.35, *p* < 0.001). Additionally, perceived teacher enthusiasm was positively related to perceived autonomy support (*r* = 0.54, *p* < 0.001) and perceived task value (*r* = 0.50, *p* < 0.001), while perceived autonomy support was positively related to perceived task value (*r* = 0.55, *p* < 0.001).

**Table 1:** Mean, standard deviation, and intercorrelations of all measures.

Variable	*M*	*SD*	1	2	3	4	5	6
(1) Boredom proneness	3.59	0.69	—					
(2) Perceived task difficulty	2.93	0.96	0.12^∗∗^	—				
(3) Perceived teacher enthusiasm	3.78	0.90	-0.17^∗∗∗^	0.01	—	—		
(4) Perceived autonomy support	3.56	0.70	-0.31^∗∗∗^	-0.01	0.54^∗∗∗^	—		
(5) Perceived task value	3.76	0.88	-0.26^∗∗∗^	0.06	0.50^∗∗∗^	0.55^∗∗∗^	—	
(6) Class-related boredom	2.22	0.84	0.37^∗∗∗^	0.28^∗∗∗^	-0.26^∗∗∗^	-0.40^∗∗∗^	-0.35^∗∗∗^	—

*N = 734; ^∗∗^*p* < 0.01, ^∗∗∗^*p* < 0.001.*

### Testing the Mediating Model

**FIGURE 2:**
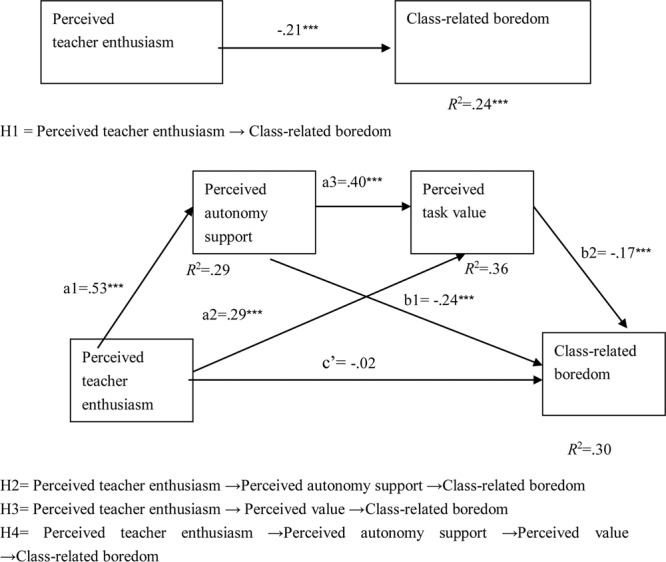
**Mediation model**.

Based on the proposed mediating model shown in **Figure 1** and the intercorrelations of all of the measures in **Table 1**, a path analysis was conducted in Mplus 7 (Muthén and Muthén, 1998–2013), to test the total effect of perceived teacher enthusiasm on class-related boredom and the three specific mediating effects. The standardized estimated path coefficients for these effects have been shown in **Figure 2**.

Firstly, after controlling for the effects of gender, age, grade, boredom proneness and perceived task difficulty, perceived teacher enthusiasm significantly predicted class-related boredom (β = -0.20, standardized β = -0.21, *p* < 0.001). Secondly, in the mediation model, after controlling for the effects of gender, age, grade, boredom proneness, and perceived task difficulty, perceived teacher enthusiasm significantly predicted perceived autonomy support (β = 0.42, standardized β = 0.53, *p* < 0.001) and perceived task value (β = 0.28, standardized β = 0.29, *p* < 0.001); perceived autonomy support significantly predicted perceived task value (β = 0.50, standardized β = 0.40, *p* < 0.001) and class-related boredom (β = -0.28, standardized β = -0.24, *p* < 0.001); and perceived task value significantly predicted class-related boredom (β = -0.17, standardized β = -0.017, *p* < 0.001).

Additionally, as shown in **Table 2**, the assessment of the indirect effects in this multiple mediator model suggested a significant indirect serial mediated effect of perceived autonomy support and perceived task value (95% CI = [-0.05, -0.02]), as well as two separate indirect effects through perceived autonomy support (95% CI = [-0.17, -0.09]) and perceived task value (95% CI = [-0.08, -0.03]) in the relationship between perceived teacher enthusiasm and class-related boredom. These results suggested that perceived autonomy support and perceived task value fully mediated the effect of perceived teacher enthusiasm on class-related boredom.

**Table 2:** Indirect effects of perceived teacher enthusiasm on class-related boredom.

Paths of indirect effect	Effect size (standardized β)	95% CI
*Perceived teacher enthusiasm →Perceived autonomy support →Class-related boredom*	0.53 × (-0.24) = (-0.13)^∗∗∗^	[-0.17, -0.09]
*Perceived teacher enthusiasm →Perceived value →Class-related boredom*	0.29 × (-0.17) = (-0.05)^∗∗^	[-0.08, -0.03]
*Perceived teacher enthusiasm →Perceived autonomy support →Perceived value →Class-related boredom*	0.53 × 0.40 × (-0.17) = (-0.04)^∗∗∗^	[-0.05, -0.02]

*N = 734. The path coefficient in the model is standardized coefficient (standardized β). ^∗∗^*p* < 0.01, ^∗∗∗^*p* < 0.001.*

## Discussion

Partially based on the control-value theory of achievement emotions (Pekrun, 2006; Pekrun et al., 2007) and the SDT (Deci and Ryan, 1987; Deci et al., 1991), the current study examined mediating models on the relationships among perceived teacher enthusiasm, perceived autonomy support, perceived task value, and class-related boredom in Chinese college students. The present findings suggest that perceived autonomy support and perceived task value may fully mediate the effect of perceived teacher enthusiasm on class-related boredom as serial and parallel inductors. These findings have implications for research on the relationship between teacher enthusiasm and students’ class-related emotions.

Perceived teacher enthusiasm consistently and indirectly predicted class-related boredom through the full mediation of perceived autonomy support and perceived task value, which provides strong evidence for the positive role that teacher enthusiasm plays in reducing the level of class-related boredom among college students. Our findings showed that although teacher enthusiasm may not predict class-related boredom significantly and directly, enthusiastic teachers may provide more autonomy support and may induce a higher task value of the course for their students, which may reduce students’ class-related boredom. As posited in the integrated model of the control-value theory of achievement emotions (Pekrun, 2006; Pekrun et al., 2007), through the mediation roles of perceived control and value, external environmental variables (such as teacher enthusiasm and autonomy support) may decrease the levels of class-related boredom in college students. A high level of perceived teacher enthusiasm and autonomy support further improves college students’ perceived task value in class-related learning, which eventually results in lower class-related boredom and other positive learning outcomes. As situational factors, teacher enthusiasm and autonomy support were found to be important predictors of class-related boredom, considering the reciprocal relationship between positive and negative emotions (Cacioppo and Berntson, 1994; Schimmack, 2001; Smith et al., 2006; Schimmack and Colcombe, 2007), which is aligned with the research results of Winberg et al. (2014) on positive emotions. Our results also showed that perceived autonomy support and perceived task value fully mediated the relationship between perceived teacher enthusiasm on class-related boredom as serial and parallel inductors, which suggests the importance of the two mediators in this process. Furthermore, for the mediating role of autonomy support between teacher enthusiasm and its outcomes, Winberg et al. (2014, p. 288) argued that teacher enthusiasm would facilitate “a relatively autonomous extrinsic type of motivation.” Similarly, the results of Ruzek et al. (2016) showed that perceived autonomy mediated the relationship between teacher emotional support and students’ engagement and motivation. In addition to the short-term class-related variables, future research should further clarify whether teacher enthusiasm and autonomy support continuously drive individuals to achieve long-term school success and make career choices.

Additionally, after controlling for the effects of perceived teacher enthusiasm, perceived autonomy support had a unique contribution to the prediction of college students’ perceived task value and class-related boredom. From the perspective of the SDT, perceived autonomy support can fulfill psychological needs, which eventually results in a higher perceived task value and other positive learning outcomes (Deci et al., 1991). In line with this, abundant research has confirmed the effects of autonomy support and its core components on learning processes and outcomes, including class-related values, emotions, and motivations (e.g., Reeve et al., 2004; Patall et al., 2008, 2010, 2014; Tsai et al., 2008; Sierens et al., 2009; Jang et al., 2010; Daschmann et al., 2011; Kaplan and Assor, 2012; Patall, 2012, 2013; Tze et al., 2014).

The findings of the current study have important practical implications. Both perceived teacher enthusiasm and perceived autonomy support serve as significant predictors of task value and can thus be used as important tools to reduce the most frequent and harmful academic experience (i.e., class-related boredom). Thus, when designing courses or interventions for reducing class-related boredom, researchers, educators, and counselors should also focus on both teacher enthusiasm and autonomy support. Related courses or activities should guide teachers to develop and provide more enthusiasm and autonomy support for their students. Colleges may also consider designing assistant programs and training sessions for teachers to promote teacher enthusiasm and related behaviors aimed at providing autonomy support to students. In turn, this would promote students’ perceived task value in the class and would reduce their class-related boredom, which would eventually result in more positive learning outcomes. According to the findings of the serial mediating role of perceived autonomy and task value between teacher enthusiasm and class-related boredom, first and most importantly, we should guide and facilitate teachers’ enthusiasm for their subjects and teaching.

Despite the theoretical and practical implications discussed above, the current study has several possible limitations. Firstly, according to the model of the control-value theory of achievement emotions, in addition to perceived teacher enthusiasm and autonomy support, teaching quality, students’ subjective control, and achievement goals also play unique roles in reducing students’ class-related boredom (Pekrun, 2006; Pekrun et al., 2007). However, these factors were not included in the current study. Future research should include these variables when examining the effects of teacher enthusiasm on students’ learning. Secondly, the current study utilized college students’ self-reporting, and it was conducted at the conclusion of one semester. The one-sided self-report answers and the upcoming final examination may have affected the level of teacher variables and class-related boredom. To test the accuracy of the mediating model, future research should attempt to overcome this limitation by measuring more teacher (or teaching) and student intraindividual variables at several time points and levels, to better estimate how teacher enthusiasm and autonomy support influence college students’ learning outcomes. Thirdly, as the current study was conducted with a sample of students from a medical college in China, whether the findings discussed above could be generalized to other college and university students remains to be determined. Lastly, the current study was conducted with reference to compulsory medical courses. Thus, further research needs to determine whether our findings could be generalized to other optional and non-medical college or university courses.

## Conclusion

This study expands upon existing knowledge regarding the relationship between teacher enthusiasm and class-related boredom, and its findings are novel and insightful, both theoretically and practically. This study not only clarifies that medical college students’ perceived teacher enthusiasm negatively predicts their class-related boredom, but it also supports the role of their perceived autonomy support and task value as mediators in this relationship. In short, the results suggest that medical college students’ perceived autonomy support and task value mediate the dampening effect of their perceived teacher enthusiasm on class-related boredom. To this end, the present study offers an important foundation for future work.

## Author Contributions

GC and MY contributed to the conception construction and design of the study and to the analysis and interpretation of data. XZ contributed to the collection of data. Also, GC wrote the drafted paper and MY made critical revisions. All authors approved the final manuscript for publication.

## Conflict of Interest Statement

The authors declare that the research was conducted in the absence of any commercial or financial relationships that could be construed as a potential conflict of interest. The reviewer BR and the handling Editor declared their shared affiliation, and the handling Editor states that the process nevertheless met the standards of a fair and objective review.
